# Report of Diseases in the Public and Private Practice of One of the Physicians of the Finsbury Dispensary, from the 20th of April, to the 20th of May

**Published:** 1805-06-01

**Authors:** J. Reid

**Affiliations:** Grenville Street, Brunswick Square


### *Report of Diseases in the public and private Practice of one of the Physicians of the Finsbury Dispensary, from the 20th of April, to the 20th of May.*

| | | | |
|---|---|---|---|
| Rheumatismus | 14 | Menorrhagia | 7 |
| Phthisis | 5 | Fluor albus | 8 |
| Catarrhus | 11 | Asthenia | 24 |
| Pneumonia | 2 | Hydrops | 9 |
| Diarrhœa | 6 | Pneumatosis | 3 |
| Dysenteria | 1 | Morbi Cutanei | 14 |
| Febres | 2 | Morbi Infantiles | 27 |
| Amenorrhœa | 16 | | Several |

Several cases have occurred lately of imaginary phthisis. Nothing is so common as to mistake simple debility, and its natural consequences, for a diseased affection of the pulmonary organs. On which account, in cases of debility, remedies are continually applied, which, instead of alleviating, are calculated merely to aggravate its degree, and to perpetuate its continuance.

The patient more frequently dies of a consumption of medicine than of a consumption of the lungs.

Two instances of fever have this last month been under the care of the Reporter, which, although alarming from its violence and duration, as well as from the constitution and previous habits of the subjects of it, have experienced relief, and have been brought into a convalescent state, by appropriate and energetic applications.

Except in cases of a natural or artificial old age, or, in other words, when the constitution has been exhausted either by intemperance or by time, no person *ought* to die of fever.

To this opinion, which the writer ventured publicly to advance, in one of his Reports, nearly six years ago, every day of his subsequent experience has afforded additional confirmation.

Where there is no mutilation of structure, the spark of life may, by careful and skilful management, be preserved, as long as there is left any fuel to support it.

An incalculable number of lives are diurnally sacrificed to an ignorance of this doctrine, or to an insufficient capacity of applying it to professional practice.

The well-being of the human race has not suffered so much even from politcal or religious, as from medical ignorance and superstition. The vulgar, and what are considered established axioms or dogmas, with regard to physic, inevitably lead either to a miserable scepticism, or to an essential and destructive depravity of judgement.

"I was (says the celebrated Dr. Brown) for the space of fifteen years engaged in endeavouring to comprehend and apply, in learning and in teaching, the doctrines of the schools. After consuming more than half this time without satisfaction, or consciousness of either instructing myself or others, I began to deplore the science of medicine as incomprehensible and uncertain; till, at length, truth flashed upon my mind, and occasioned the same delightful feelings which the dawn of day produces on a wandering wandering and benighted traveller—*quasi prima diurna lux demum adfulsit**."

J. REID.

*Grenville Street, Brunswick Square, May* 26, 1805.

* To the author of this article, few circumstances of his life have afforded a higher degree of satisfaction, than the share he has had in the education of a number of intelligent students and incipient practitioners of medicine, who, he flatters himself, have left him with minds impregnated with a knowledge, and penetrated with a conviction of the truth and importance of the Brunonian system, which, although not without its alloy of error, is the only one that, in its fundamental principles, rest upon the firm and indestructible basis of a genuine phoilsophy.

